# Redox processes acidify and decarboxylate steam-pretreated lignocellulosic biomass and are modulated by LPMO and catalase

**DOI:** 10.1186/s13068-018-1159-z

**Published:** 2018-06-18

**Authors:** Ausra Peciulyte, Louise Samuelsson, Lisbeth Olsson, K. C. McFarland, Jesper Frickmann, Lars Østergård, Rune Halvorsen, Brian R. Scott, Katja S. Johansen

**Affiliations:** 1 Division of Industrial Biotechnology, Department of Biology and Biological Engineering, Chalmers University of Technology, Kemivägen 10, 412 96 Gothenburg, Sweden; 2 Novozymes Inc., 1445 Drew Ave, Davis, CA 95618 USA; 3 Novozymes North America, 77 Perry’s Chapel Church Road, Franklinton, NC 27525 USA; 4 Novozymes A/S, Krogshøjvej 36, 2880 Bagsværd, Denmark; 5 Department of Geosciences and Natural Resource Management, Copenhagen University, Rolighedsvej 23, 1958 Frederiksberg, Denmark

**Keywords:** Hydrogen peroxide, pH/proton activity, Biorefinery, Decarboxylation, Enzymes, Wheat straw

## Abstract

**Background:**

The bioconversion of lignocellulosic feedstocks to ethanol is being commercialised, but further process development is required to improve their economic feasibility. Efficient saccharification of lignocellulose to fermentable sugars requires oxidative cleavage of glycosidic linkages by lytic polysaccharide monooxygenases (LPMOs). However, a proper understanding of the catalytic mechanism of this enzyme class and the interaction with other redox processes associated with the saccharification of lignocellulose is still lacking. The in-use stability of LPMO-containing enzyme cocktails is increased by the addition of catalase implying that hydrogen peroxide (H_2_O_2_) is generated in the slurry during incubation. Therefore, we sought to characterize the effects of enzymatic and abiotic sources of H_2_O_2_ on lignocellulose hydrolysis to identify parameters that could improve this process. Moreover, we studied the abiotic redox reactions of steam-pretreated wheat straw as a function of temperature and dry-matter (DM) content.

**Results:**

Abiotic reactions in pretreated wheat straw consume oxygen, release carbon dioxide (CO_2_) to the slurry, and decrease the pH. The magnitude of these reactions increased with temperature and with DM content. The presence of LPMO during saccharification reduced the amount of CO_2_ liberated, while the effect on pH was insignificant. Catalase led to increased decarboxylation through an unknown mechanism. Both in situ-generated and added H_2_O_2_ caused a decrease in pH.

**Conclusions:**

Abiotic redox processes similar to those that occur in natural water-logged environments also affect the saccharification of pretreated lignocellulose. Heating of the lignocellulosic material and adjustment of pH trigger rapid oxygen consumption and acidification of the slurry. In industrial settings, it will be of utmost importance to control these processes. LPMOs interact with the surrounding redox compounds and redirect abiotic electron flow from decarboxylating reactions to fuel the oxidative cleavage of glycosidic bonds in cellulose.

**Electronic supplementary material:**

The online version of this article (10.1186/s13068-018-1159-z) contains supplementary material, which is available to authorized users.

## Background

Decomposition of plant material in nature is driven primarily by the action of microbial enzymes capable of deconstructing the complex structure of the plant cell wall. In addition to enzymes, abiotic (non-enzymatic) reactions driven by redox processes also play an important role [1]. An important contribution to abiotic decomposition of organic matter involves reactive oxidants produced by Fe(II) oxidation. This pathway is likely one of the mechanisms through which organic compounds are oxidized under acidic conditions [2]. Moreover, dissolved organic matter has been shown to be an integral component in biogeochemical electron transfer reactions due to its ability to facilitate redox reactions [3].

Agricultural and forestry residues are the primary lignocellulosic feedstocks being used to produce ethanol in commercial facilities. The biomass is usually subjected to chemical and/or physical pretreatment, followed by enzymatic hydrolysis and ethanol fermentation. Interestingly, the industrial saccharification of lignocellulose takes place under conditions that resemble those in the natural environment in peat bogs. This ecological niche is characterised by fluctuating concentrations of oxygen in the upper strata of the bog. A few centimetres below the secondary water table, the oxidative process consumes the dissolved oxygen at a rate higher than the passive influx from the air.

Industrial saccharification of lignocellulose relies on highly optimised enzyme cocktails for the efficient release of monosaccharides from the pretreated feedstock. Lytic polysaccharide monooxygenases (LPMOs), a group of enzymes discovered in more recent years [4], are often included in such enzyme cocktails and are particularly important drivers of cellulose hydrolysis performance. LPMOs are Cu-containing enzymes that require oxygen and a source of electrons to cleave glycosidic bonds [5, 6]. Importantly, certain LPMOs initiate cellulose degradation by cleaving internal β1,4-glycosidic bonds without prior hydration (de-crystallisation) of individual cellulose chains. In doing so, LPMOs create access points for processive exo-cellulases [7]. Therefore, to achieve efficient saccharification, it is important that oxygen and appropriate electron donors are present [8–10]. Recent work suggests that activated oxygen in the form of H_2_O_2_ may serve as the co-substrate for LPMO catalytic cleavage of polysaccharides [11–13] rather than or in addition to dioxygen. This scheme is discussed in the context of the results generated in this paper in “Discussion”.

Lignocellulose is rich in phenolic compounds as industrial pretreatments, such as acid and neutral steam explosion, only partially disrupt and solubilize the lignin fraction. These phenolic compounds serve as electron donors for LPMO reactivity during saccharification, and in many cases, no exogenous reducing agent is required [6, 14, 15]. The LPMO-activating properties of many individual phenolic compounds have been tested [10, 16–18]. The redox potential of the electron donors is critical for the LPMO reactivity [6, 17].

**Fig. 1:**
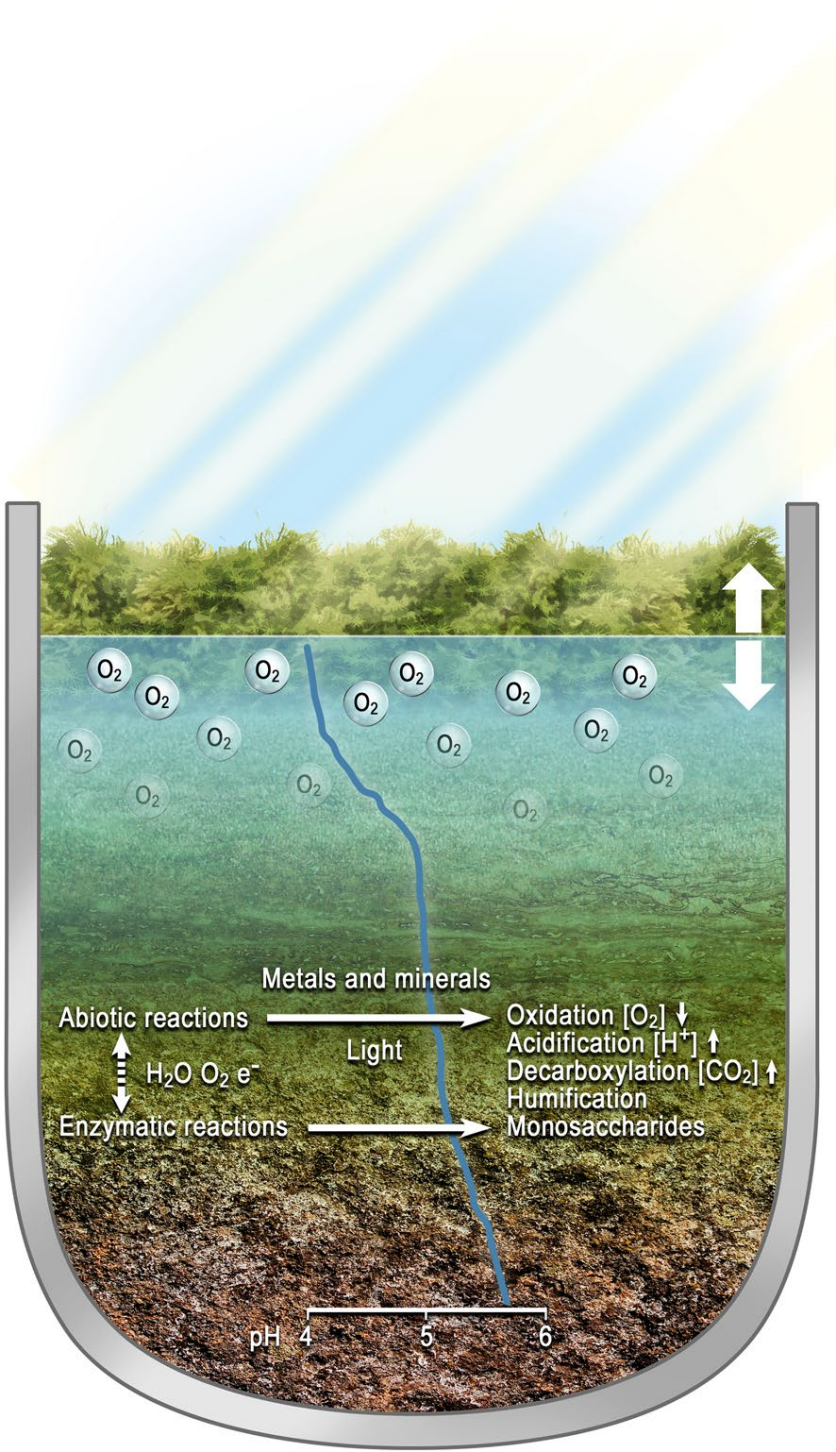
Illustration of the principal reactions involved in the decomposition of plant residues in the environment. The abiotic reactions are catalysed by transition metals such as iron, and by minerals of geological origin. Light promotes certain abiotic reaction paths. Enzymes secreted into the environment by microbes such as saprotrophic bacteria and fungi catalyse a similar range of reactions, but via different chemical mechanisms and at different rates. A representative pH profile based on [47] is indicated

The oxygen content of pretreated lignocellulosic material used in industrial saccharification has not been described systematically, but will be highly process- and configuration dependent. However, the interaction between the enzymatic and the abiotic oxygen-consuming redox reactions is complex and largely unexplored. This is illustrated in Fig. 1.

In nature, both the microbial decomposition processes involving extracellular enzymes and abiotic reactions are inhibited by acidic and reduced-oxygen conditions. The dissolved oxygen level is also an important parameter for optimal enzymatic hydrolysis of lignocellulose. A low level of dissolved oxygen (2% saturation from the air) is reportedly advantageous for the saccharification of pretreated wheat straw when compared to further reduced-oxygen conditions achieved by purging the reaction with nitrogen gas [18]. However, oxidative inactivation of commercial cellulase mixtures is a significant factor influencing the overall saccharification efficiency under ambient oxygen conditions, and the addition of catalase has been shown to reduce the inactivation rate of cellulase mixtures [9].

In stirred-tank bioreactors, such as those used in industry and in pilot-scale laboratories, the pH is automatically adjusted to a value determined by the optimum pH for the enzyme cocktail. Prior to this study, the amount of base titrant required during the saccharification of lignocellulose was highly variable and dependent on the concentration of dissolved oxygen, as described below. Surprisingly, the addition of catalase dramatically reduced the requirement of base titrant at 2% oxygen saturation of the slurry [19]. It has been assumed that enzymatic hydrolysis reactions by acetyl xylan esterases present in the enzymatic cocktail caused the lowering of the pH as a result of acetic acid released from hemicelluloses [20]. However, this hypothesis does not explain the oxygen-dependent nature of the acidification of lignocellulosic material.

The aim of this study was to gain new insights into the abiotic and enzymatic redox reactions taking place during the saccharification of pretreated lignocellulose and their dependence on the reaction conditions. We investigated the effect of the redox enzymes, LPMO and catalase, on these abiotic oxidative processes and vice versa. The composition of headspace gas samples was determined, and the change in pH in the saccharification slurry was monitored. Moreover, the effect of H_2_O_2_ delivery to the saccharification mixture by means of aldose oxidase or by adding H_2_O_2_ was studied. The aldose oxidase from *Microdochium nivale* (MnAO*x*) [21], an AA7 enzyme that can oxidize several mono-, oligo-, and polymeric saccharides and in the process transfers electrons to molecular oxygen, was used for generating H_2_O_2_ in situ.

## Results

### Requirement of base titrant in stirred-tank reactor trials

As indicated above, including a dissolved oxygen level of 2% saturation (with full saturation with the air being 100%) was advantageous for the saccharification of steam-pretreated wheat straw due to the oxygen-dependent activity of the LPMO included in the enzyme cocktail [19]. However, this level of dissolved oxygen also led to almost a doubling of the base titrant required to maintain the pH at the set value of 5.0 during incubation (Table 1). The findings of this pilot study also demonstrated that the addition of catalase together with 2% oxygen saturation clearly reduced the amount of titrant required, and substantially improved the sugar yield after 5 days of incubation.

**Table 1:** Data on the combined effect on sugar release and titrant requirement for a controlled level of oxygen saturation and the addition of catalase during saccharification of steam-pretreated wheat straw at 20% DM content. Reproduced with permission from [16]

	Glucose (g/L)		Xylose (g/L)		25% NaOH titration (mL)
Incubation time	3 days	5 days	3 days	5 days	5 days
N_2_-flushed	45.2	49.5	23.3	23.6	4.9
2% O_2_	61.4	63.3	24.9	25.1	9.6
2% O_2_ + catalase	60.2	67.8	24.9	26.3	5.7

It was clear from the above data that there is no direct coupling between the amount of titrant required and the sugar yield. This observation was substantiated in stirred-tank saccharification experiments with DM contents of 12, 14, 16, and 18% (Additional file 1: Table S1A), and incubation temperatures of 46, 48, 50, and 52 °C (Additional file 1: Table S1B).

### The abiotic component of pH decrease

**Fig. 2:**
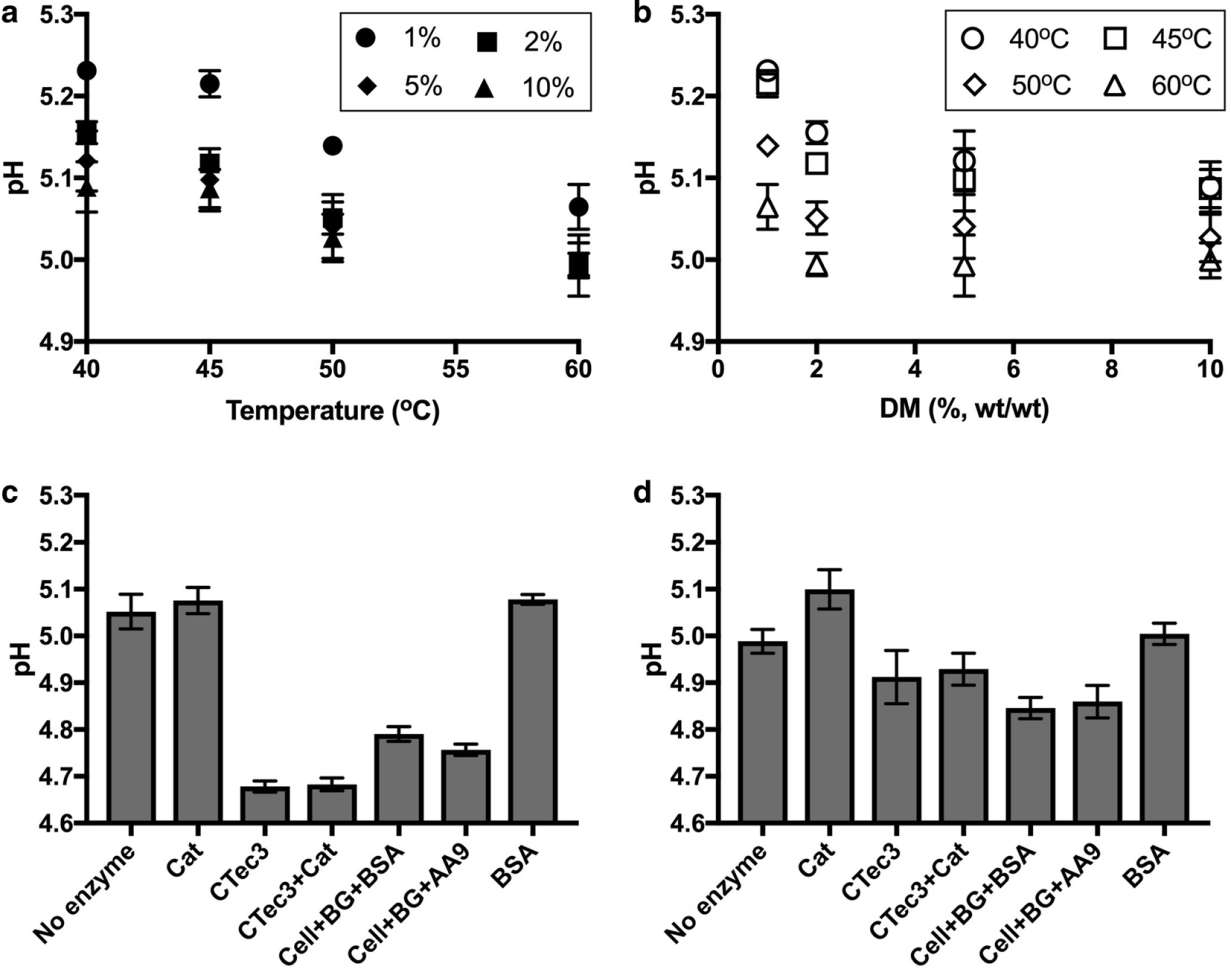
Change in pH of pretreated wheat straw slurry during 24 h of incubation. **a** Effect of incubation temperature and **b** effect of DM content in the absence of enzymes. Steam-pretreated wheat straw was diluted to DM contents of 1, 2, 5, or 10% (wt/wt), the pH was adjusted to 5.3, and the samples incubated at 40, 45, 50, or 60 °C. **c**, **d** Show the effect of enzyme addition to pretreated wheat straw slurry at 10% DM incubated at 50 °C. The data shown in (**d**) are the result of re-adjusting the pH to 5.0–5.1 and continuing incubation for an additional 24 h. The values given are the average of 3–12 replicates, and the error bars represent the standard deviation. *No enzyme* no addition of enzymes, *Cat* catalase; CTec3: Cellic^®^ CTec3; Cell: Celluclast 1.5L; BG: β-glucosidase; BSA: bovine serum albumin; AA9: TaAA9A

To study the factors underlying the decrease in pH (increase in H^+^ activity) of pretreated lignocellulose in greater detail, steam-pretreated wheat straw slurry was first incubated at four different DM concentrations (1, 2, 5, and 10% (wt/wt)) and four different temperatures (40, 45, 50, and 60 °C), for 24 h in sealed serum bottles without the addition of any enzymes. Prior to the incubation, the pretreated wheat straw slurry had a pH at room temperature of 3.6. This increased slightly upon dilution of the slurry with tap water to DM contents of 1, 2, 5, and 10% (Additional file 1: Figure S1A). The requirement of KOH per gram of DM to adjust pH to 5.3 increased as DM concentration increased (Additional file 1: Figure S1B). The pH of the slurry as a function of DM and temperature after incubation is shown in Fig. 2a, b.

A strong correlation was observed between the decrease in pH and increasing incubation temperature. The effect of DM content on the pH of the slurry is clear at lower DM concentrations of 1 and 2%. At 5 and 10% DM, the differences in pH are small. Acidification of the slurry at 5 and 10% DM may be limited by oxygen or redox-scavenging effects of lignin [22] and sugars [23, 24]. Nonetheless, it is evident that in the cases investigated, the pH of the slurry decreased during incubation without added enzymes in the presence of oxygen. For example, at 10% DM content and 50 °C, the pH decreased from 5.3 to 5.0 in 24 h due to abiotic reactions in the lignocellulosic slurry.

### The enzymatic component of acidification during saccharification

To investigate whether LPMO and catalase activity affected the amount of base titrant required during saccharification, enzyme mixtures with and without supplemental AA9 from *Thermoascus aurantiacus* (TaAA9) were used under ambient and reduced-oxygen conditions. Celluclast^®^, which is a wild-type *Trichoderma reesei* whole-enzyme mixture, was used as an LPMO-poor cellulase cocktail. Mixtures were prepared containing 80% Celluclast^®^ (by protein mass) and 10% β-glucosidase (BG) to ensure rapid and complete conversion of cellobiose to glucose, thereby avoiding product inhibition of the cellobiohydrolases and simplifying product quantification. The remaining 10% of the mix consisted of either TaAA9 or inert protein in the form of BSA. Furthermore, the effects of an LPMO-rich cellulose cocktail Cellic^®^ CTec3 (referred to hereafter as CTec3) and *Thermoascus aurantiacus* catalase were evaluated. All slurry samples had acidified after 24 h (Fig. 2c) and after pH adjustment and further 24 h of incubation (Fig. 2d). Cellulase cocktails had a strong acidifying effect on pH, whereas TaAA9 had no clear effect. Catalase addition to the slurry had only a minor effect on pH under the conditions tested here.

### Can acetic acid release explain the acidification?

**Fig. 3:**
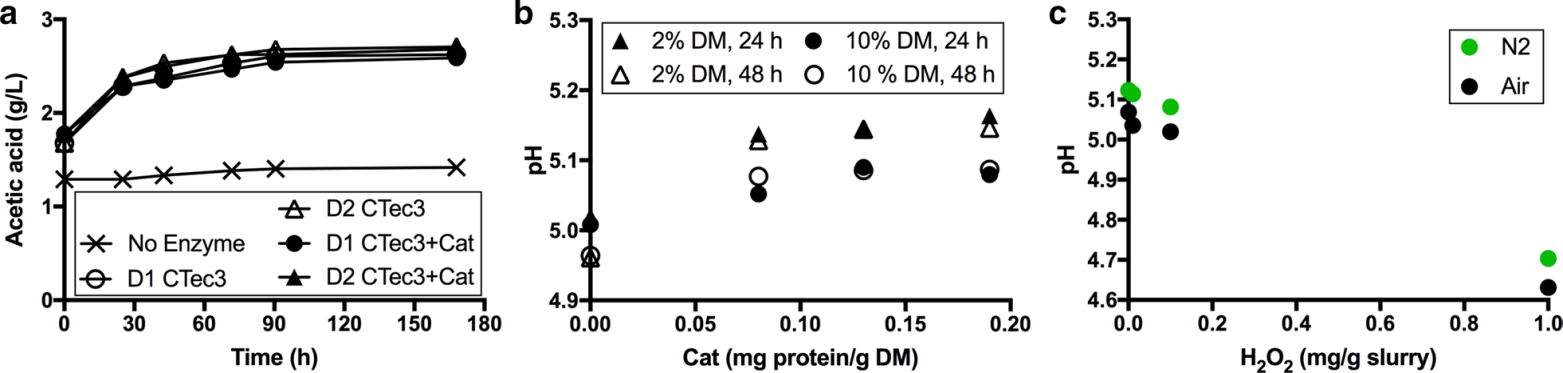
Acetic acid release and pH in steam-pretreated wheat straw slurry adjusted to pH 5.3 during incubation at 50 °C. **a** Acetic acid released during the saccharification of 10% DM wheat straw slurry. The CTec3 loading was 2.8 (D1) or 8.4 (D2) mg total protein/g cellulose. The catalase loading was 0.08 mg protein/g DM (approximately 11,000 U/ml slurry). **b** Addition of catalase reduces the decrease in pH during 24 and 48 h incubation of 2% DM, and 10% DM incubated for 24 and 48 h. **c** Effect of added H_2_O_2_ (to a concentration of 2.9 and 29 mM) on the pH of 10% DM wheat straw slurry after 24 h incubation at 50 °C under ambient air and reduced-oxygen conditions. The values given are the average of duplicate samples, and the error bars represent the standard deviation. *Cat* catalase, *CTec3* Cellic CTec3

The acetic acid concentration in wheat straw samples with and without enzyme treatment was quantified to investigate whether acetic acid release could explain the observed acidification. The samples with the pH ranging from 3.6 to 5.3 were analysed by HPLC. In this pH range, the samples contained a mixture of dissociated form (acetate) and undissociated form of acetic acid. During the analysis, the pH of the eluent was 2, and at this pH, almost 100% of acetic acid molecules exist in undissociated form. The 10% slurry contained about 1.4 g/L acetic acid, and this concentration remained almost constant throughout 172 h of incubation at 50 °C in the absence of enzymes (Fig. 3a). This is in contrast to the change in pH from 5.3 to 5.0 during 24 h of incubation (Fig. 2c). The addition of CTec3 with or without catalase resulted in a gradual increase in acetic acid concentration. The release of acetic acid was initially rapid, but levelled off resulting in approximately 2.6 g/L acetic acid at both high and low CTec3 concentrations. The presence of catalase had no effect on acetic acid concentration (Fig. 3a).

To further study the pH-stabilising effect of catalase, pretreated wheat straw was adjusted to pH 5.3 after dilution with tap water to 2 and 10% DM (as before). Three doses of catalase (0.08, 0.13, and 0.19 mg/g DM) were added. After 24 h of incubation, the slurry had acidified in all cases (Fig. 3b). In the absence of catalase, both 2 and 10% DM slurry samples acidified from pH 5.3 to 5.0. The drop in pH was reduced in the presence of catalase, as the final pH was approximately 5.13 in the 2% DM samples and pH 5.08 in the 10% DM samples. For a correct comparison of pH values, all the samples were brought to room temperature prior the measurement.

### Does the addition of H_2_O_2_ cause a decrease in pH?

To better understand the effects that H_2_O_2_ may have on this system, CTec3 was incubated with pretreated wheat straw with and without four additions of 1 mg H_2_O_2_/g slurry, i.e., approximately 29 mM H_2_O_2_. The first dose of H_2_O_2_ was added after 18 h of pre-incubation to liquefy the lignocellulosic material. In the absence of catalase, the addition of H_2_O_2_ increased the amount of KOH required from 135 to 230 µL during 120 h of incubation (data not shown). This is equivalent to a 60% increase in KOH requirement. An initial addition of 0.08 mg/g DM catalase to the reaction prevented the acidification caused by H_2_O_2_ addition, as evidenced by a titrant requirement of 135 µL (data not shown). As reported previously [9], the glucose yield was reduced by the addition of H_2_O_2_ in a concentration-dependent manner, and the effect was more pronounced under reduced-oxygen conditions. A similar H_2_O_2_-dose-dependent decrease in pH after 24 h of incubation was seen in the absence of enzymes (Fig. 3c). It is, therefore, likely that H_2_O_2_ is involved in the oxidative acidification of the slurry.

### Does enzymatic H_2_O_2_ generation cause a decrease in pH and glucose yields?

**Fig. 4:**
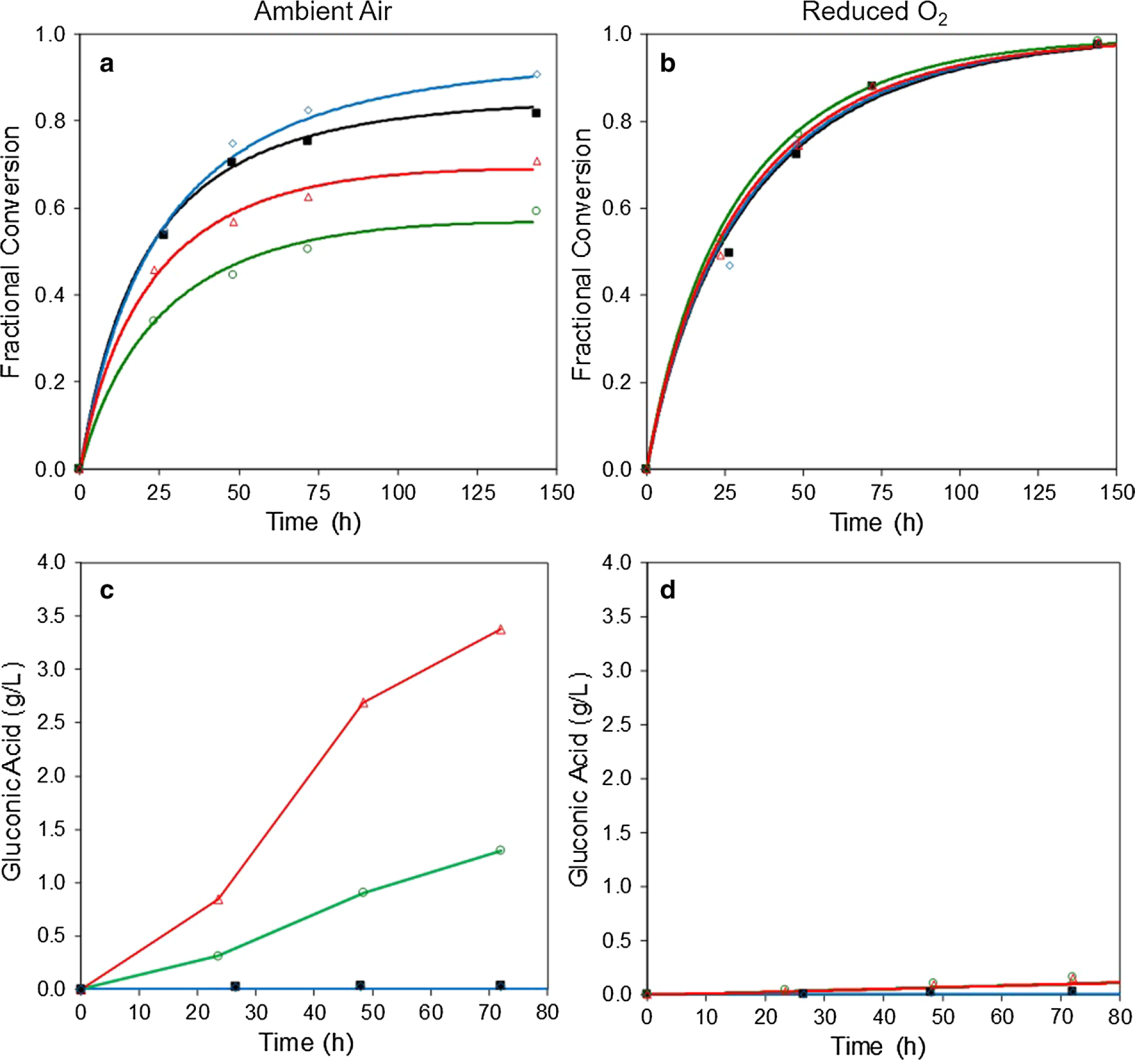
Effect of catalase on cellulose hydrolysis in the presence and absence of MnAO*x* and reduced oxygen. Pretreated wheat straw was incubated at 50°C, pH 5 for 96 h under conditions of ambient air (approximately 20% O_2_) or reduced oxygen (approximately 0.3% O_2_). The enzyme cocktail consisted of CTec3 (8.4 mg protein/g cellulose) and 0.22 mg/g cellulose catalase (blue diamonds), CTec3 + 0.22 mg/g cellulose MnAO*x* + catalase (red triangles), CTec3 + MnAO*x* (green circles), or CTec3 alone (black squares). **a**, **b** Show the fractional conversion of cellulose to glucose, while **c**, **d** Show the concentrations of gluconic acid. The curves shown in **a**, **b** are model fits

The effects of in situ-generated H_2_O_2_ on biomass acidification and glucose yields were then investigated by incubating CTec3 with steam-pretreated wheat straw in the presence and absence of MnAO*x*. These experiments were carried out under both ambient air and reduced-oxygen conditions and in the presence and the absence of exogenous catalase. Graphs of cellulose conversion to glucose and gluconic acid under each of these conditions are shown in Fig. 4. These data were analysed using the kinetic models described in Additional file 2: Figures S2 and S3, to help evaluate the combined effects of MnAO*x*, oxygen, and catalase on glucose and gluconic acid production.

The addition of catalase to CTec3 increased glucose yields after 24 h of incubation in ambient air (Fig. 4a). Model fits to these progress curves suggest that catalase decreased the inactivation rate constant of CTec3, thereby increasing the half-life of the enzyme cocktail from 36 to 76 h (Table 2). The addition of catalase had no significant effect on the catalytic rate constant of CTec3. Both these observations are consistent with those in a previous study [9]. An overlay of these progress curves with those published previously is shown in Additional file 2: Figure S4.

**Table 2:** Estimated values of the catalytic (*k*_s_) and inactivation (*k*_*i*_^a^) rate constants for CTec3 in the presence and the absence of MnAO*x* and catalase

	*k*_s_ (g h^−1^g^−1^)	*k*_i_^a^ (10^−2^ g h^−1^g^−1^)	*t*_1/2_ (h)	2 M KOH (µL)
Ambient air				
Control	15.4 ± 1.6	1.95 ± 0.4	36 ± 7	120 ± 0
Cat	14.5 ± 1.5	0.91 ± 0.3	76 ± 27	130 ± 0
MnAO*x*	6.3 ± 1.9*	1.67 ± 0.8	42 ± 20	400 ± 28
Cat + MnAO*x*	11.7 ± 4.4	1.60 ± 0.6	43 ± 17	473 ± 4
Reduced-oxygen				
Control	11.6 ± 1.6	n/a**	–	100 ± 0
Cat	11.3 ± 1.7	n/a**	–	100 ± 0
MnAO*x*	13.5 ± 2.2	n/a**	–	110 ± 0
Cat + MnAO*x*	12.5 ± 1.4	n/a**	–	110 ± 0

The CTec3 cellulose hydrolysis progress curves shown in Fig. 4a, b were fit using the model shown in Additional file 2: Figure S2. The values estimated for *k*_s_ and *k*_*i*_^a^ are shown below. Moreover, the amount of 2 M KOH required to adjust the pH to 5.0 is shown

* P value < 0.05 versus CTec3 control under ambient air

** Estimated parameter value is indistinguishable from zero

The addition of MnAO*x* decreased the extent of cellulose conversion to glucose in ambient air within the first 24 h (Fig. 4a). The formation of gluconic acid accounted for a relatively small fraction of the total glucose equivalents produced (Fig. 4c). This suggests that MnAO*x* reduced cellulose conversion to glucose via a detrimental effect on CTec3 or on the pretreated wheat straw, rather than solely by producing oxidized glucose equivalents as alternative reaction products. This effect occurred early in the incubation and is reflected in the kinetic model as a decrease in the catalytic rate constant of CTec3 from 15.4 to 6.3 g h^−1^ g^−1^ (Table 2), while the inactivation rate constant remained constant. The combined addition of catalase and MnAO*x* markedly improved the conversion of cellulose to glucose when compared to the addition of MnAO*x* alone. Interestingly, gluconic acid concentrations also increased approximately threefold (Fig. 4c). When considering glucose and gluconic acid levels together, cellulose conversion measured in the presence of catalase and MnAO*x* was similar to the CTec3-only control (Additional file 2: Figure S5).

The addition of catalase may have increased gluconic acid concentrations by stabilising MnAO*x* under these assay conditions. This is reasonable if the H_2_O_2_ produced as a co-product by MnAO*x* contributes to the oxidative inactivation of this enzyme as shown previously [25]. In degrading H_2_O_2_, catalase reduces the inactivation rate of MnAO*x*. Alternatively, catalase may increase the apparent catalytic rate of MnAO*x* by producing oxygen in situ. If true, this would indicate that oxygen availability limits the activity of MnAO*x* in the absence of catalase, at least at some stages of the reaction. This is considered further in Additional file 2: Figure S6 and Table S4.

### Pronounced acidification of the slurry was observed in the presence of MnAO*x*

The pH of each saccharification reaction above was adjusted to 5.0 using a 2 M solution of KOH at each sampling time and the total volume of KOH added is listed in Table 2. The total volume of KOH required to adjust the pH to 5.0 increased from 120 µL in the absence of MnAO*x* to 400 µL when it was added under the ambient air condition, while the glucose yield decreased. When catalase was included, in addition to MnAO*x*, the volume of KOH increased further to 473 µL and glucose yield increased. Therefore, as discussed above, the KOH requirement is not directly correlated to the glucose yield. The amount of titrant required is not a simple function of the concentration of gluconic acid produced, as can be seen when comparing the conditions with and without catalase. After 72 h of incubation in the presence of catalase, the molar titrant requirement relative to the molar production of gluconic acid was approximately 3:1, whereas it was approximately 6:1 in the absence of catalase. This provides further evidence that H_2_O_2_ contributes to acidifying reactions during the decomposition of lignocellulosic material.

### Pretreated wheat straw consumes oxygen

**Fig. 5:**
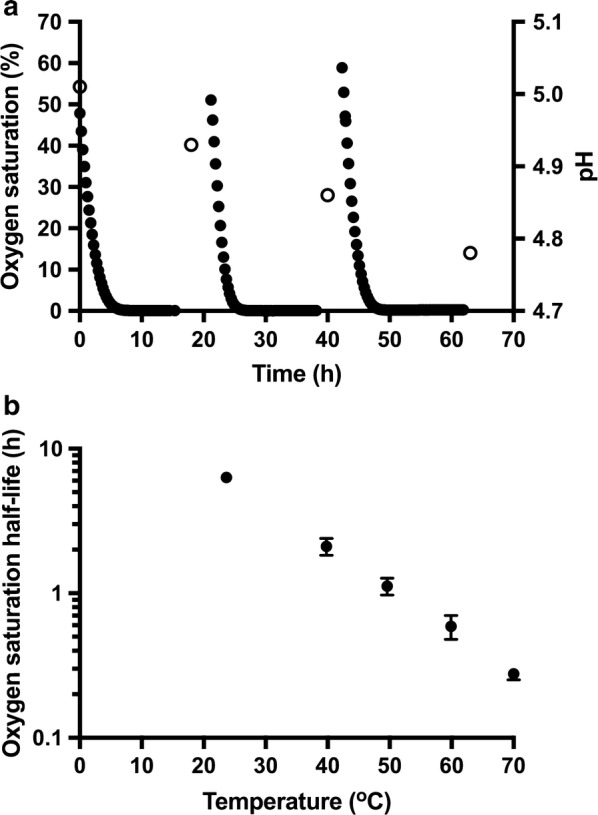
**a** Oxygen saturation in pretreated wheat straw slurry during three rounds of incubation at 50 °C (filled circles). The slurry was adjusted to pH 5.0 prior to the initial incubation, and was noted after each round of incubation (empty circles). **b** Dependence of the half-life of the oxygen saturation on the temperature. Error bars show the standard deviation of the calculated half-live of oxygen for replicates at each temperature, with a singlet value at 23 °C

The temperature-dependent rate of abiotic oxygen depletion in wheat straw slurry was determined in samples incubated without any stirring and with ambient air in the headspace using fluorescence-based sensors [26]. During incubation of the biomass at 50 °C, the oxygen content decreased to < 0.5% oxygen saturation (Fig. 5a). Here, 100% oxygen saturation means that the slurry is completely saturated with the air. A gradual acidification from pH 5.0 to pH 4.8 was observed by the end of each incubation cycle.

The change in oxygen saturation during incubation between 24 and 70 °C is well described as an exponential decay, with a linear correlation of the temperature and the oxygen half-life (Fig. 5b). For one experiment at 70 °C, the headspace gas was not exchanged with fresh air between cycles, and the headspace oxygen content was also measured through three cycles of heating, each with drop of slurry DO, followed by cooling, measurement of headspace  %oxygen, mixing of biomass and headspace gas, and reheating. Starting at 95%, headspace  %oxygen dropped to 74, 63 and 54% at the end of each cycle, while the slurry dropped to < 1% DO. Based on the change in oxygen content of the headspace, it is estimated that 18 μmol oxygen was consumed during each cycle of incubation at 70 °C and 50 µmol/g DM/24 h. This is a considerably larger number than the 4 µmol oxygen expected to be soluble in the biomass at the start of incubation (Additional file 2: Table S4). Although the oxygen saturation in the biomass falls to < 0.5% during incubation, the oxygen content of the headspace remained high, demonstrating that the diffusion rate into the biomass from the headspace is lower than the rate of oxidation of biomass components.

### Decarboxylation reactions take place at industrially relevant saccharification conditions

When running biomass saccharification experiments in stirred-tank reactors, it is common to observe the formation of air bubbles in the slurry (Additional file 1: Figure S2) during the incubation. To determine if the composition of the gases in the headspace of the reactor changes throughout the saccharification reaction, its chemical composition was determined using GC analysis. The gas pressure in the samples was also recorded in a parallel series of samples.

**Fig. 6:**
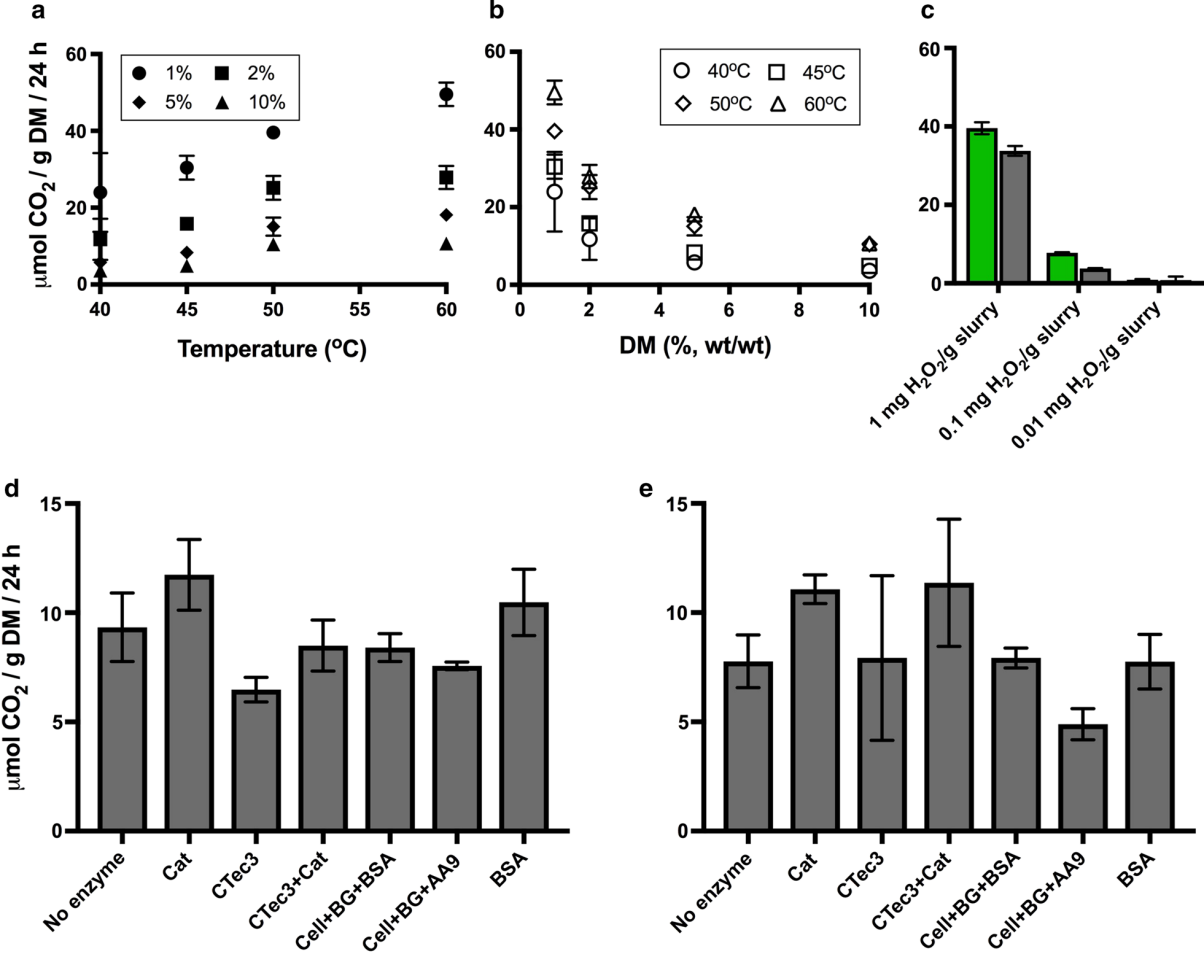
Production of CO_2_ during incubation of pretreated wheat straw adjusted to pH 5.3. **a** Effect of temperature, and **b** DM content, during incubation for 24 h. DM contents of 1, 2, 5, and 10%, at 40, 45, 50, and 60 °C. The values given are the average of four replicates, and the error bars represent the standard deviation. **c** Dosage-dependent production of CO_2_ during the incubation of 10% DM pretreated wheat straw slurry with H_2_O_2_ at 50 °C for 24 h under ambient atmosphere (grey bars) or reduced-oxygen conditions (green bars). The CO_2_ produced in samples without the addition of H_2_O_2_ has been subtracted. **d** Effect of enzymes on CO_2_ production during the first 24 h at 50 °C after initial pH adjustment to 5.3 and **e** during the following 24 h after pH re-adjustment to 5. *No enzyme* no addition of enzymes, *Cat* catalase, *CTec3* CelliC CTec3, *BSA* bovine serum albumin, *AA9* TaAA9

A considerable increase in the concentration of CO_2_ (up to 2.5%) compared to ambient air (0.0355%) was immediately evident. From the results presented in Fig. 6, there is a positive correlation between temperature in the industrially relevant range of 40–60 °C and the concentration of CO_2_ in the headspace. The concentration of CO_2_ also increased with the DM content from 1 to 10% (wt/wt), but the CO_2_ produced per gram of DM decreased.

The effect of enzymes on CO_2_ release was studied by the addition of cellulolytic enzyme cocktails, as described above. The CO_2_ produced during the first 24 h of incubation was about 9 µmol/g DM in the absence of enzymes, with inert protein (BSA) and with Celluclast^®^ + BG. The addition of CTec3 reduced the CO_2_ concentration in the headspace compared with slurry alone and with BSA (Fig. 6d). However, the addition of catalase led to increased CO_2_ concentration in the headspace when added to the slurry in the absence of other enzymes. This effect is highly surprising in light of the H_2_O_2_-dependent CO_2_ production, as shown in Fig. 6c and discussed below. Catalase in combination with CTec3 also resulted in a higher CO_2_ concentration than CTec3 alone, and a value close to the CO_2_ produced in the slurry alone. Incubation for another 24 h resulted in similar relative concentrations of CO_2_ for the different conditions (Fig. 6e). The combination of Celluclast^®^ and AA9 clearly resulted in the lowest CO_2_ concentration, while CTec3 alone and together with catalase gave more variable results, leading to large standard deviations.

### H_2_O_2_ addition leads to increased decarboxylation

To determine whether H_2_O_2_ can be directly linked to CO_2_ production, we investigated whether the addition of three different amounts of H_2_O_2_ would change the CO_2_ profile of the gas in the headspace after 24 h of incubation at 50 °C. The addition of 0.01 mg H_2_O_2_/g slurry had no effect on CO_2_ production, whereas the addition of 0.1 mg/g slurry led to an approximate doubling of the CO_2_ production, compared to the slurry alone. The addition of 1.0 mg H_2_O_2_/g slurry, i.e., 29 mM H_2_O_2_, led to a striking increase in the concentration, to 4 mM (40 µmol/g DM) CO_2_, which is about 400 times the concentration in the air (Fig. 6c).

## Discussion

The ubiquitous balancing act between the generation and elimination of reactive oxygen species is well known [27]. Tightly controlled coupling of oxygen consumption with hydrocarbon oxidation can be found in enzymes such as soluble methane monooxygenase, but is a major challenge in biocatalysis [28]. With the introduction of LPMOs in commercial cellulase cocktails, this is true also for industrial conversion of lignocellulose to ethanol [9]. Dissolved oxygen and electron donors with appropriate redox potential are required for LPMO activity. However, oxygen is unavoidably consumed by abiotic routes during degradation of organic matter [2]. In the present study, we found that such abiotic reactions in the saccharification slurry led to the acidification and decarboxylation of steam-pretreated wheat straw during incubation at industrially relevant conditions of pH and temperature. These chemical processes seem to be modulated by the presence of the oxido-reductases catalase and LPMO.

### Oxidative acidification

It has been found that 2% dissolved oxygen saturation led to almost a doubling of the base titrant required to maintain the pH at 5.0 for the duration of enzymatic saccharification of pretreated wheat straw, compared with the reduced-oxygen condition. It was also found that catalase could mitigate the titrant requirement (Table 1). The relationship between the oxidation and the acidification of decomposing plant material is seldom described in the biotechnological literature. We, therefore, investigated the effect of generic process parameters such as temperature and DM content on the acidification of steam-pretreated wheat straw.

### H_2_O_2_ addition increases acidification

The mitigating effect of catalase on the requirement of base titrant was unexpected. The mechanism of catalase involves reduction by H_2_O_2_ of the active site Fe(III) of the enzyme. This is followed by proton/hydrogen abstraction and the release of water and oxygen, where both oxygen atoms originate from the same H_2_O_2_ molecule [29]. In other words, the catalase reaction per se is neutral, and does not explain the pH-stabilising effect of catalase. It is possible that H_2_O_2_ is an intermediate in the acidifying reactions and that removal of H_2_O_2_ by catalase stabilised the pH. Such a mechanism is supported by the observed decrease in pH due to both in situ-generated (Table 2) and exogenously added H_2_O_2_ (Fig. 3).

However, acidification by H_2_O_2_ has no straightforward chemical explanation. In acidic solutions, such as those encountered during the saccharification assays described here, H_2_O_2_ acts as a strong oxidizing agent, leading to the consumption of protons. In basic solutions, H_2_O_2_ is a reductant and protons are released. The reducing property of H_2_O_2_ in combination with lignocellulose has been observed previously by Banerjee et al. [30], who used alkaline H_2_O_2_ to pretreat corn stover and noted a downward shift in pH. However, at pH values around 5, H_2_O_2_ could be participating in chemical reactions with other compounds in the mixture, thereby indirectly causing the pH to fall.

### H_2_O_2_ effect on saccharification

It was found that in situ H_2_O_2_ generation by aldose oxidase addition (under the conditions used) was detrimental to the saccharification process (Fig. 4a). The glucose concentration was reduced by about 1/3 when the oxidase was present, and under these conditions, catalase can substantially, but not completely, alleviate this negative effect. The magnitude of the observed effect of MnAO*x* on glucose yields within the first 24 h is striking. This implies that one or more substrates for MnAO*x* are available early in the reaction, despite the relatively low cellulose conversion. Similarly, the low levels of gluconic acid suggest that substrates other than glucose or cello-oligomers, such as cellobiose, are used. The specificity of MnAO*x* from *M. nivale* to different sugars, including xylose, has been reported [21, 31]. Using a *Coprinus cinereus* peroxidase-coupled assay based on the rate of O_2_ consumption, Xu et al. [21] determined that a recombinant MnAO*x* had *K*_M_ of 42 and 59 mM, and *k*_cat_ of 4 and 12 s^−1^, on glucose and cellobiose, respectively, at 40 °C. In addition, the relative activity of MnAO*x* on xylose (38% of the activity on cellobiose) was only slightly lower than the activity reported on glucose (50%). It is plausible that the high initial rate of MnAO*x* activity is due, at least in part, to the utilization of soluble sugars, including xylose and xylo-oligomers, from the pretreatment liquor.

It is unlikely that the oxidized glucose per se has a negative effect on the saccharification efficiency, as catalase improves the accumulation of both glucose and gluconic acid (Fig. 4c). The enzyme-stabilising effect of the removal of H_2_O_2_ by catalase, as discussed previously [9, 25], is in agreement with this observation.

Recent findings propose that H_2_O_2_ can be a co-substrate (in place of dioxygen) in LPMO catalysis of polysaccharides cleavage in the presence of small amounts of reducing agent [11]. The kinetics of such reaction were studied by Kuusk et al. [12] and the molecular mechanism of such a reaction was studied by QM/MM calculations [13, 32]. Due to the presence of endogenous reducing capacity in pretreated biomass, H_2_O_2_ could serve as a co-substrate for the LPMO catalytic cycle on this material. However, with the limited number of experimental conditions used in the present study, no evidence of a positive effect of added H_2_O_2_ is seen. What is clear is that H_2_O_2_ will react with lignocellulosic material to increase H^+^ activity and produce CO_2_. Several publications discussing the relevance of O_2_ versus H_2_O_2_ as LPMO co-substrate have appeared in the literature during the final stage of the review process of the current work [33–35], but no data for lignocellulosic material have been presented. The applicability of process configurations involving the addition of H_2_O_2_ for industrial saccharification of lignocellulose warrants still further investigation.

### Abiotic decarboxylation

We observed an increase in CO_2_ concentration of up to 100 times in the headspace above slurry samples after 24 h of incubation at 50 °C (Fig. 6). To the best of our knowledge, decarboxylation reactions have not previously been discussed in the context of industrial enzymatic saccharification of lignocellulose. However, Miles and Brezonik [1] observed significant production of CO_2_ by oxygen-consuming reactions in natural humic-coloured waters. In these samples, the oxygen consumption increased with increasing DM content, with increasing pH, and with the total concentration of iron in the sample.

Abiotic, oxygen-dependent CO_2_ release from pyrogallol in deionized distilled water has also been observed [36]. This process was increased by the addition of manganese, iron or aluminium oxides. Fe(III)-containing mineral nontronite has also been found to increase CO_2_ release from the phenolic compounds pyrogallol, catechol, and hydroquinone [37]. The iron-oxide-catalysed reaction led to a final pH of 3.6 when the samples were incubated in the air and 4.2 under a nitrogen atmosphere; both of which were much lower than the initial pH of 6 of the experiment. The pretreated wheat straw used in the present study contained approximately 200 mg iron/kg of washed solids. It is likely that this content of iron (and other transition metals) in the material contributes to the abiotic acidifying and decarboxylating reactions.

### Abiotic drivers of decarboxylation

This study was carried out on the lignocellulosic substrate steam-pretreated wheat straw. As a result of the release of organic acids during hydrothermal treatment, the material had a pH of 3.5–3.9 before adjustment of the DM content and pH of the slurry [38]. When temperature and pH was increased to moderate values used in the present study, significant abiotic reactivity of the pretreated material was evident by rapid consumption of oxygen (Fig. 5). It has been shown that pH scales linearly with the logarithm of the oxygen consumption in humic water samples [1], which substantiates our observation. The difference in H^+^ activity is 25-fold between pH 3.9 (stock biomass) and 5.3 (saccharification conditions), and this will certainly change the equilibrium of the chemical reactions taking place in the slurry at the start of incubation.

The addition of H_2_O_2_ to the slurry under both ambient and reduced-oxygen conditions resulted in a dosage-dependent reduction in pH (Fig. 3c) and concomitant production of CO_2_ (Fig. 6c). Ruff degradation of aldonic acids yielding aldoses with one less carbon atom and CO_2_ [39] is a possible explanation of this observation. However, in the absence of other enzymes, catalase reduces the decrease in the pH of the slurry during incubation while increasing the CO_2_ production. Ruff degradation driven by abiotic peroxide is, therefore, not sufficient to explain the oxidative acidification and decarboxylation of pretreated wheat straw during incubation at 50 °C.

### Enzymatic contribution to decarboxylation

Catalase enhances CO_2_ production, even in the absence of other enzymes (Fig. 6d, e). One possible explanation of the enhancing effect of catalase on CO_2_ production is that it is linked to the relatively slow phenol oxidase activity of the catalase itself [40]. Phenol oxidase activity on lignin-derived compounds may lead to a quinone intermediate that is prone to decarboxylation, as this has been reported for tyrosinase-catalysed decarboxylation of 3,4-dihydroxymandelate [41]. Oxidized and reduced quinones have been found ubiquitously in dissolved organic matter in environmental samples [42]. Oxidative decarboxylation of lignin-related benzoic acids by *Aspergillus flavus* [43] and by *Pycnoporus cinnabarinus* [44] has also been reported.

However, it should be noted that both catalase and H_2_O_2_ separately increased CO_2_ production (Fig. 6c, d), while the effect was to increase (Fig. 3b) and decrease pH (Fig. 3b, c), respectively. In contrast to catalase, LPMO reduced the CO_2_ production (Fig. 6d, e). We can thus exclude the decarboxylation of carboxylic acids by LPMO as the source of CO_2_ in the incubated samples. Such a reaction is catalysed by lactate oxidase, which is a flavin-containing monooxygenase [45]. Perhaps, the extent of decarboxylation leading to increased CO_2_ in this system is related to the availability of dissolved oxygen. Catalase and H_2_O_2_ addition both have the common effect of generating oxygen in situ via either the enzyme-mediated or spontaneous conversion of H_2_O_2_ to dioxygen and water. Similarly, LPMOs utilize dioxygen as a co-substrate and will have the effect of reducing dissolved oxygen concentrations.

**Fig. 7:**
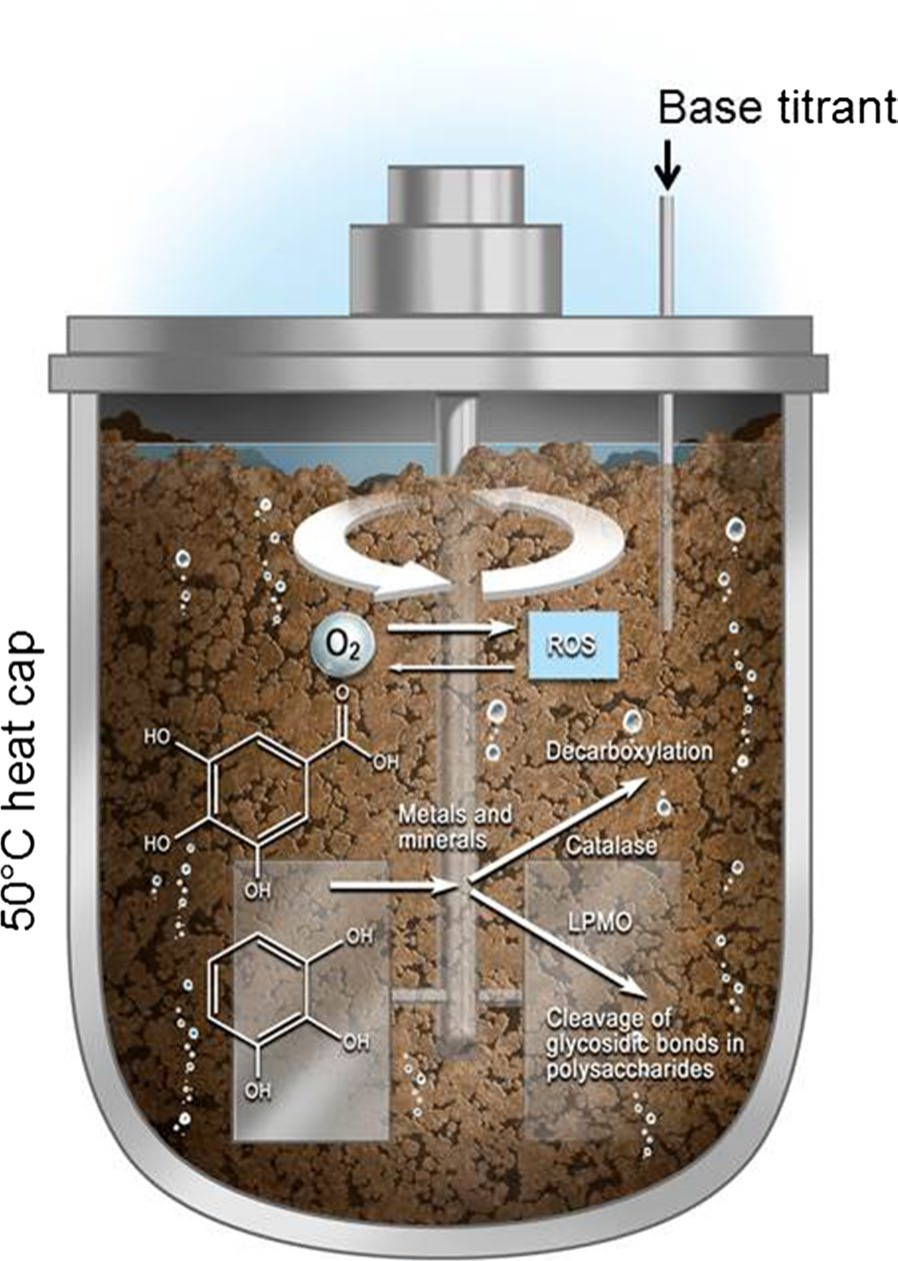
Illustration of potential reaction paths for the critical redox processes during decomposition of lignocellulosic biomass. Protic electron-donating compounds present in the material, such as gallate and pyrogallol, contribute to both abiotic and enzymatic reactions

Taken together, and illustrated in Fig. 7, these observations suggest that the redox enzymes redirect the electron flow in the lignocellulosic slurry.

### CO_2_ release affects pH

An increase in CO_2_ concentration of up to 100-fold in the lignocellulosic slurry has a strong effect on pH. For example, a rough calculation shows that 2% CO_2_ in pure water has a pH of 4.4. The pH range in the pretreated biomass slurry was similar to the pH of some humic-rich water samples [1] and to the pH in the surface layers of *Sphagnum* peat bogs [46]. Peat bogs are characterised by surface layers that are hydrologically isolated from the influence of groundwater and surface water and from mineral matter in the sediments. Analysis of bog pore water samples has revealed that dissolved CO_2_ strongly influenced the pH of samples, where the partial pressure of CO_2_ was not in equilibrium with the atmosphere. For example, a water sample taken at a depth of 2 m had a pH of 5, but after sparging the sample with N_2_ gas, the pH increased to 7.5. In such samples, organic acids and dissolved CO_2_ are equally important acids in determining the pH [47]. The situation arising during industrial saccharification of lignocellulose thus appears to resemble that in a peat bog. Our preliminary results suggest that the principles discussed here for steam-pretreated wheat straw are general and apply to other lignocellulosic materials including wood.

In bio-industrial settings, the control of pH is critical and most commercial cellulases, including CTec3 used in this study, have a pH optimum around 5.

We observed a more pronounced catalase-related reduction in titrant consumption in stirred-tank reactors set up with a constant air (gas) flow, than in assays using sealed tubes or vials. Catalase addition combined with the removal of CO_2_ by air exchange could be helpful in minimizing the need for base titrant while maintaining a certain level of dissolved oxygen during incubation. If the amount of NaOH consumed in 2 L stirred-tank reactors is extrapolated to a full-scale lignocellulosic ethanol factory (Additional file 1: Table S2), it is clear that the daily requirement of dry NaOH amounts to several tons. The significance of reducing this is evident, both in terms of economy and in terms of logistics. Moreover, the addition of NaOH will result in the formation of a range of salts, such as NaCl and Na_2_CO_3_, which require proper disposal. It is, therefore, of great importance in industry to limit the acidification of the lignocellulosic slurry as much as possible, even when such reactions do not directly lower the monosaccharide yield.

## Conclusions

Controlled oxygen saturation in the reaction slurry is necessary during enzymatic saccharification of pretreated lignocellulose in industrial processes. A consequence of the oxic condition is abiotic reactions that lead to the acidification and decarboxylation of the lignocellulosic material. Addition of H_2_O_2_ as direct or indirect co-substrate of LPMOs gives similar effects. It will be important to control these processes in industrial settings.

As illustrated in Fig. 7, the redox enzymes change the course of such abiotic chemical reactions in the slurry. Most importantly, catalase stabilises the pH through a mechanism that could involve phenol oxidase activity of the enzyme or micro-oxygenation of the slurry. The result is likely to be increased decarboxylation of aromatic acids. Removal of CO_2_ by exchanging the headspace air may result in a more stable pH of the reaction slurry.

These observations may also be relevant for the decomposition of plant material in the environment as a reminder that CO_2_ production is not exclusively via respiration.

## Methods

### Substrates, enzymes, and reagents

All reagents were laboratory grade, unless otherwise stated. Two batches of steam-pretreated wheat straw slurry (kindly prepared by Mats Galbe, Lund University, Sweden) were used in the experiments (Additional file 2: Tables S1 and S2). The slurry (denoted SS-2014-00041) was the same as that used previously [9], and was received frozen, stored at − 20 °C, and thawed prior use. The solid composition of the insoluble pretreated solid fraction and the soluble pretreatment liquor are shown in Additional file 2: Tables S1 and S2. This material was used as whole slurry in all the experiments except those involving carbon dioxide measurements. The slurry from another batch using the same pretreatment was used in the experiments to measure carbon dioxide, and was stored in darkness at + 4 °C. The commercial enzyme products Cellic^®^ CTec3 and Celluclast^®^ 1.5L and the following experimental enzymes were obtained from Novozymes A/S. The aldose oxidase used in this study was cloned from *Microdochium nivale* (Genbank Accession BD103535) and expressed from *Fusarium venenatum* [21]. Cloning and expression of the *Thermoascus aurantiacus* (Ta) AA9 (Accession ABW56451) were performed as previously described [48]. Catalase from *Thermoascus aurantiacus* (Accession DD046677) was expressed in *Aspergillus niger* and purified as described previously [49]. The properties of this enzyme were in accordance with previously published data [50] e.i.a calculated molecular weight of 75 kDa, *K*_m_ was 48 mM, and *K*_cat_ was 137,000 s^−1^ (absorbance at 240 nm, 10 mM H_2_O_2_ in 50 mM phosphate buffer pH 7.0 at 30 °C and unites defined as amount of enzyme that decompose 1 µmol H_2_O_2_/min). The β-glucosidase from *Aspergillus fumigatus* (Accession EAL88289) was cloned and expressed from *Aspergillus oryzae* as described elsewhere [51]. All the experimental enzymes were more than 90% pure. Bovine serum albumin (BSA) (98% Sigma-Aldrich) was used as an inert protein. Enzymes and BSA aliquots were stored at − 20 °C until used. Enzyme stock samples were prediluted with tap water prior to addition to the slurry samples. Tap water was also used for the dilution of wheat straw slurry. H_2_O_2_ solution (≥ 30%, Sigma-Aldrich) was used in the indicated reactions.

The iron content of the pretreated wheat straw was determined by in-house inductively coupled plasma optical emission spectrometry (ICP-OES) analysis at Novozymes and found to be approximately 200 mg/kg solid and 30 mg/kg liquid.

### Lignocellulose incubation with and without enzymes

During saccharification in the presence and the absence of MnAO*x* in ambient air and reduced-oxygen conditions, pretreated wheat straw was incubated with CTec3, loaded at 8.4 mg total protein/g cellulose, and the addition of catalase 0.22 mg/g cellulose (approximately 11.000 U/ml) as described previously [9] unless otherwise stated. MnAO*x* was also loaded at 0.22 mg/g cellulose. Please see Additional file 2 for further details regarding the kinetics. The initial concentration of total dry matter was 10% w/w, resulting in an initial cellulose concentration of 4% w/w. The initial combined concentration of xylose and xylo-oligomers in the liquid portion of the pretreated substrate was 19.6 g/L.

For carbon dioxide (CO_2_) measurements, experiments were performed using 15 g reactions in 30 mL serum bottles capped with airtight butyl rubber caps (Rubber B. V., The Netherlands, part No.: 7395), and sealed with aluminium crimp caps (with a removable centre) prior to incubation. Mixing was performed on an orbital shaker at 180 rpm. The pH was initially adjusted to 5.3 with 1 M KOH. Four DM concentrations (wt/wt), and 1, 2, 5, and 10% were investigated at temperatures of 40, 45, 50, and 60 °C for 24 h without the addition of enzymes under ambient air conditions. H_2_O_2_ (from ≥ 30% stock solution) was added to the slurry preparations at 10% DM to give final loadings of 0.01, 0.1, and 1 mg H_2_O_2_/g of slurry, and the bottles were then capped and incubated for 24 h at 50 °C. The same experiments, with the addition of H_2_O_2_, were performed under reduced-oxygen conditions. Wheat straw slurry at 10% DM, and pH 5.3, was flushed with N_2_ for 15 min prior to placing the substrate in a disposable Atmosbag (Sigma-Aldrich) flushed with N_2_. Inside the Atmosbag, the substrate was aliquoted to serum bottles, and H_2_O_2_ was added prior to sealing the bottles. All the reactions with enzymes were performed in ambient air conditions. The effect of enzymes was investigated on 10% DM slurry at 50 °C, by loading enzyme blends according to protein mass, consisting of 80% Celluclast^®^ 1.5L, 10% BG and 10% AA9 (or 10% BSA instead of TaAA9), or 100% CTec3, as described previously [9]. The total protein dose in each experiment was 4.9 mg protein (not including catalase)/g DM. Catalase was loaded at 0.08 mg/g DM. After 24 h incubation, gas was sampled from the headspace and the pressure was measured (as described below). The bottles were opened and the pH was re-adjusted to 5 when the pH fell below 5. The serum bottles were then sealed and incubation continued for an additional 24 h. Control reactions were performed with catalase loadings of 0.08, 0.13, and 0.19 mg as enzyme active protein on 2 and 10% DM slurry. The samples were incubated at 50 °C for 48 h and measurements were made in the same manner after 24 h as described above, except that the pH was not adjusted. During pH measurements, the samples were mixed and the bottles were kept open in ambient air for about 1 h.

### GC analysis of CO_2_ in the headspace

The gas samples collected from the headspace were incubated in serum bottles sealed with airtight butyl rubber caps. A stopcock was mounted on the syringe, and gas samples of 3.5–4.0 mL were collected, by piercing the septum of the cap with the needle. The stopcock was closed after sample withdrawal. Care was taken not to allow the samples to cool during gas collection. The syringe containing the gas sample was then left to cool for 2 min at room temperature. The plunger of the syringe was then depressed until resistance was felt, prior to injection into a two-channel gas chromatography (GC) instrument (490 Micro GC, Agilent Technologies Sweden AB), which was equipped with a thermal conductivity detector. Channel 1 was equipped with a 10 m-long Molsieve 5 column, and helium was used as the carrier gas (at a pressure of 5.44 atm), allowing for the analysis of methane, O_2,_ and nitrogen. Channel 2 was equipped with a 10 m-long CP-PoraPLOT U column, argon was used as the carrier gas (5.44 atm), and was used for the analysis of CO_2_. This column also allows for the analysis of methane. A backflush time of 13 s, an injector temperature of 110 °C, and a column temperature of 80 °C were applied. None of the headspace air samples contained hydrogen or methane at levels above the detection level of the GC equipment. The CO_2_ calibration curve was obtained using gas standards with mole fractions (vol/vol) of 10, 30, and 100% CO_2_ (all of purity over 99.99%). The number of moles (*n*) of gas in the samples was calculated according to the ideal gas law:

$$n = \frac{PV}{RT}.$$

The pressure (*P*) in the headspace was measured using a digital manometer (GMH 3111) and a relative pressure sensor (MSD 2,5 BRE; Greisinger Electronic, Regenstauf, Germany). Pressure measurements were performed after incubation by piercing the septum of the butyl rubber cap of the serum bottles.

### Consumption of O_2_

Steam-pretreated wheat straw was used as whole slurry in experiments to measure the oxygen use during incubation without mixing. Samples of biomass were adjusted to pH 5 and 10% total solids (%TS) prior to use. Percent dissolved oxygen (DO) of the biomass slurry was measured by sensors (optodes) mounted inside gas impermeable reaction tubes (Corex, USA 25 mL model 8445, 30.5 mL total volume), with fluorescent quantitation of oxygen by Fibox4 (Presens, Germany). pH was measured by Orion 8175BNWP probe on Accumet XL600 meter (Fisher Scientific, USA), with temperature compensation.

16 g of 10% total solid biomass whole slurry was placed in tubes and then gassed with the air until the DO was > 80% (100% is defined as saturated with the air). Tubes were sealed with gasketed caps and oxygen impermeable Saran Wrap (Asahi Kasei, Japan) and incubated in a water bath without mixing at setpoints of 20, 40, 50, 60, and 70 °C. DO of the substrate slurry was measured at 15 min intervals until the slurry DO was minimal, usually < 0.5%. The tubes were then placed in a room temperature circulating water bath until chilled to < 23 °C. Tubes (except 70 °C experiment) were then opened, the slurry measured for pH, the headspace gas replaced with fresh air, and the slurry mixed well to oxygenate the biomass until the slurry DO was > 80% as measured at 20–23 °C. Then, the tubes were returned to the incubation temperature (20–70 °C) for measurement of DO. This could be repeated at least four times without apparent change in oxygen use rates. The headspace DO was also measured for the samples incubated at 70 °C, without exchanging the headspace with fresh air. DO measurements attained during aeration and pH procedures when temperatures were below setpoints were not graphed or fitted.

DO measurements corresponding to timepoints within 0.5 °C of the temperature setpoints were fitted as to *N*_0_ (initial oxygen %), C the steady-state final DO, and *λ* (the decay rate) in an exponential decay formula (F1) by minimizing total pairwise error using the Microsoft Excel add-in Solver with the GRG method. Reaction rates can be described by half-life = ln(2)/*λ*; *N*(*t*) represents the predicted %DO at time *t*:

F1 $$N(t) = (N_{0} - C)\text{e}^{ - \lambda t} + C.$$

### Determination of glucose, xylose, and acetic acid

The saccharified samples were analysed using HPLC with a Rezex ROA-Organic Acid H+ (8%) column with a 3 mm I.D. (Phenomenex Inc.) maintained at 80 °C with a Carbo-H4 guard cartridge (Phenomenex Inc.) maintained at room temperature. As eluent, 5 mM H_2_SO_4_ was used at a flow rate of 0.8 mL/min in isocratic mode. Analytes were detected with a refractive index detector.

## Additional files

**Additional file 1.** Abiotic reactivity of steam pretreated wheat straw.

**Additional file 2.** Substrate composition and kinetic modelling of the saccharification process.

## Abbreviations

BG: β-glucosidase
BSA: bovine serum albumin
Cat: catalase
Cell: Celluclast^®^ 1.5L
CTec3: Cellic^®^ CTec3
DM: dry matter
GC: gas chromatography
HPLC: high-performance liquid chromatography
ICP-OES: inductively coupled plasma optical emission spectrometry
LPMO: lytic polysaccharide monooxygenase
MnAO*x*: aldose oxidase from *Microdochium nivale*
*t*_1/2_: enzyme performance half-life
TaAA9: LPMO from *Thermoascus aurantiacus* which belongs to auxiliary activity family 9
wt/wt: weight per weight
