# Supplementary material for: Redox processes acidify and decarboxylate steam-pretreated lignocellulosic biomass and are modulated by LPMO and catalase
Source: Biotechnol Biofuels. 2018 Jun 18;11:165. doi: 10.1186/s13068-018-1159-z (PMC6004669; doi:10.1186/s13068-018-1159-z)
Supplement: Supplementary file 1 — Additional file 1. Abiotic reactivity of steam pretreated wheat straw. [file 13068_2018_1159_MOESM1_ESM.docx]

Additional file 1


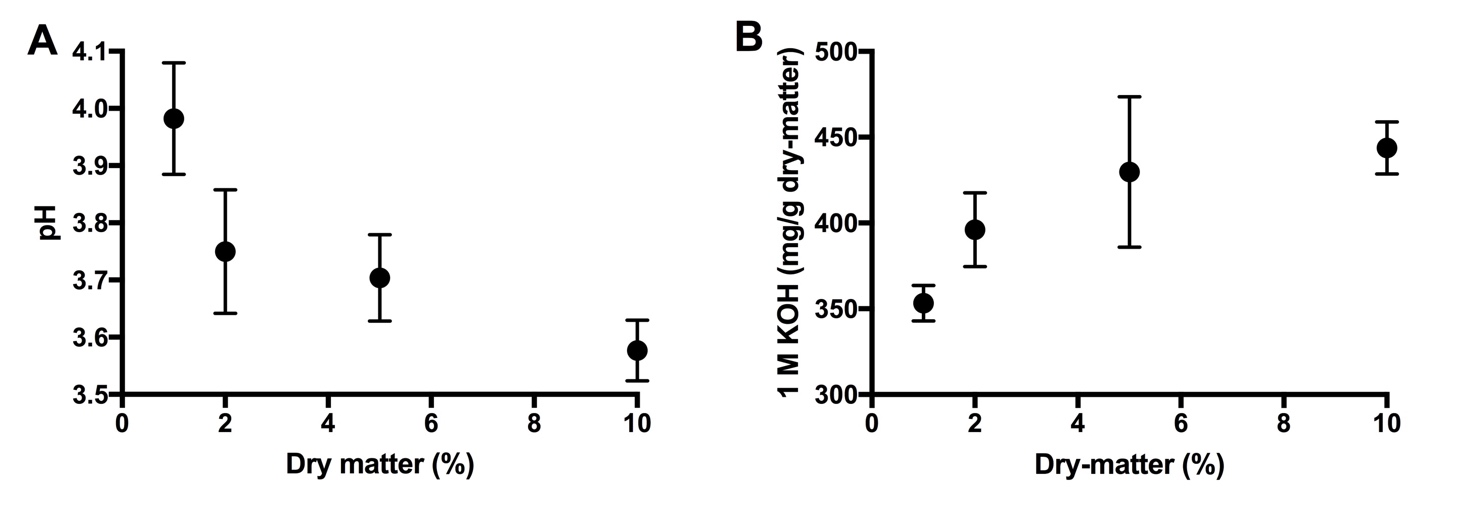


**Figure S1.** pH values after wheat straw slurry was diluted with tap water to a certain % of dry-matter (A). Amount (mg) of 1 M KOH required to adjust pH to 5.3 for wheat straw slurry of different % of dry-matter (B). Values are reported as the average values of two up to nine replicates and the error bars represent standard deviation.


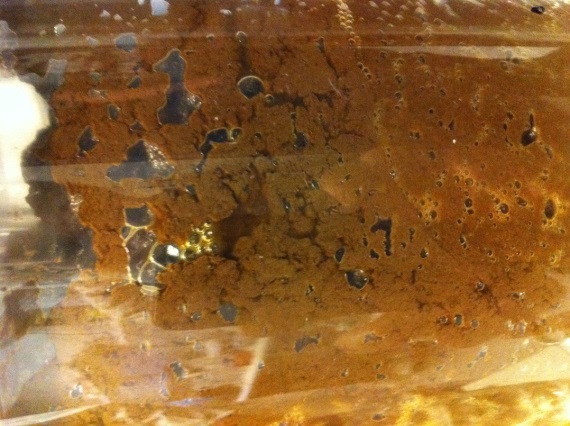

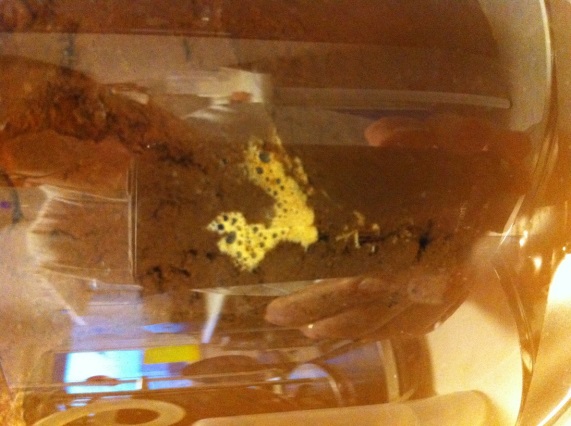


**Figure S2**. Representative pictures of air bubbles formed in preatreated wheat straw slurry during early stages of saccharification. Infors stirred tank reactor at 50°C.

**Table S1A:** The consumption of base is proportional to the dry matter content of the biomass slurry during enzymatic saccharification at 50°C, pH 5.0 in 2L Infors stirred-tank reactors. The glucose yield is also proportional to the dry matter content and the addition of KOH.

| DM [%] | 12 | 14 | 16 | 18 |
| --- | --- | --- | --- | --- |
| KOH [ml] | 11.2 | 13.9 | 15.6 | 18.7 |
| Glucose yield [%] | 57.0 | 51.2 | 46.8 | 44.3 |

**Table S1B:** The consumption of base is proportional to the temperature of the biomass slurry during enzymatic saccharification at 50°C, pH 5.0. The glucose yield is not proportional to the temperature and addition of KOH.

| Temp | 46°C | 48°C | 50°C | 52°C |
| --- | --- | --- | --- | --- |
| KOH [ml] | 10.3 | 19.9 | 21.1 | 27.8 |
| Sugar, 122h [g/l] | 46 | 44 | 50 | 46 |

**Table S2:** Extrapolation of the use of base during enzymatic saccharification of pretreated wheat straw at commercial scale.

|  | laboratory stirred tank reactor | | Commercial scale saccharification | |
| --- | --- | --- | --- | --- |
| Capacity | 2 | Kg | 3000 | T/day |
| NaOH use, no Catalase | 9.6 | ml 25% | 14400 | L 25%/day |
|  | 3.1 | g dry wgt | 4597 | kg dry wgt/day |
| NaOH us, with Catalase | 5.7 | ml 25% | 8550 | L 25%/day |
|  | 1.8 | g dry wgt | 2730 | kg dry wgt/day |
