# Supplementary material for: Redox processes acidify and decarboxylate steam-pretreated lignocellulosic biomass and are modulated by LPMO and catalase
Source: Biotechnol Biofuels. 2018 Jun 18;11:165. doi: 10.1186/s13068-018-1159-z (PMC6004669; doi:10.1186/s13068-018-1159-z)
Supplement: Supplementary file 2 — Additional file 2. Substrate composition and kinetic modelling of the saccharification process. [file 13068_2018_1159_MOESM2_ESM.docx]

Additional file 2

# Substrate composition

The composition of the insoluble solids fraction of the steam pretreated wheat straw used in this study is shown below in Table S1. Most of the xylan was solubilized during pretreatment while glucan remained primarily in the insoluble solids fraction. Similarly, the concentrations of individual chemical species in the liquid fraction of the wheat straw slurry after pretreatment are shown in Table S2.

**Table S1**: Composition of the insoluble solids fraction of the steam pretreated wheat straw.

| Fraction | Concentration  % (w/w) |
| --- | --- |
| Glucan | 41.9 |
| Xylan | 4.7 |
| Arabinan | 0.3 |
| Klason Lignin | 30.4 |
| Ash | 7.3 |

**Table S2: Composition of the soluble solids in the liquid fraction of the steam pretreated wheat straw.**

| Fraction | Concentration  (g/L) |
| --- | --- |
| Glucose | 0.2 |
| Gluco-oligomers | 4.8 |
| Xylose | 1.9 |
| Xylo-oligomers | 25.6 |
| Arabinose | 1.1 |
| Lactic acid | 0.3 |
| Formic acid | 0.5 |
| Acetic acid | 1.8 |
| HMF | 0.1 |
| Furfural | 0.7 |


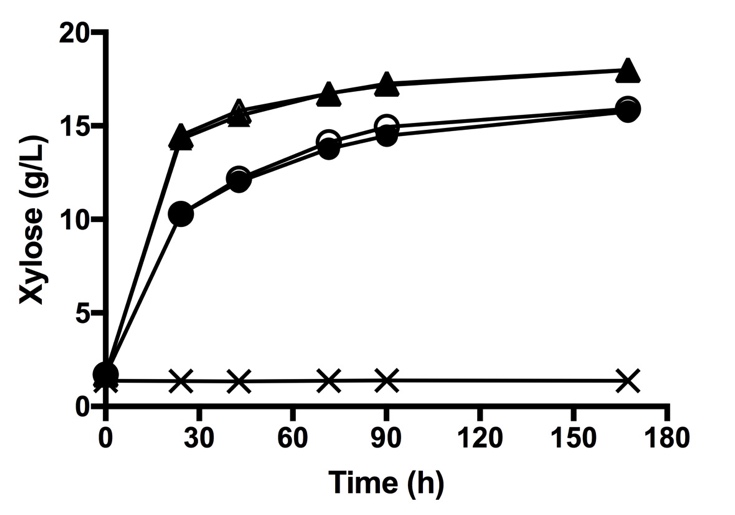


**Figure S1.** The xylose release progress curves during the saccharification of 10% dry-matter wheat straw slurry. The enzyme loading was 5 mg/g DM CTec3 sample (empty circle), the 5 mg/g DM CTec3+catalase (filled circle), 15 mg/g CTec3 (empty triangle), 15 mg/g DM CTec3+catalase (filled triangle), and no enzyme (crosses). Catalase was loaded at 80 µg/g DM. Values are reported as the average of duplicate and the error bars represent standard deviation.

# Kinetic modelling

**Figure S2**: Two stage Michelis-Menten kinetic model of cellulose hydrolysis with competitive product inhibition and cellulase inactivation (Scott et al., 2015). A model describing the conversion of cellulose (S) to cellobiose (G2) catalyzed by active cellulase enzyme (E_a_) is shown in panel A. The cellulase in this model is subject to competitive inhibition by both glucose (G) and cellobiose (G2) and time-dependent inactivation to E*. The model process by which cellobiose is converted to glucose (G) by β-glucosidase (Bg) is shown in panel B. The β-glucosidase is subject to competitive inhibition by glucose

A 2 stage kinetic model described previously by Scott et al., (2015) was used as the framework for analyzing the cellulose hydrolysis progress curves (Figure 4). Briefly, all of the components in the commercial cellulase tested here, except for β-glucosidase(s), contributed to the formation of cellobiose. In this model, cellulases (E) and β-glucosidase (Bg) were assumed to follow Michaelis-Menten kinetics. It was also assumed that the cellulases were subject to competitive inhibition by glucose (K_G_^a^) and cellobiose (K_G2_) and subject to first-order inactivation (*k*_i_^a^) to the inactive form, E*. β-glucosidase was subject to competitive inhibition by glucose (K_G_^b^).

The mechanism of cellulose hydrolysis has been described using Michealis-Menten kinetics with product inhibition by others (Reviewed by Sousa et al., 2011). However, model certainly does not take into account many other factors that have been reported previously to affect the rate of cellulose hydrolysis. This model was employed because it provided a suitable framework for interpreting the progress curves generated here under a limited range of substrate concentrations and enzyme doses. Complexities, such as enzyme adsorption onto cellulose (Scheiding et al., 1984; Philippidis et al., 1992; Nidetzky et al., 1993) and increasing recalcitrance of the substrate (Drissen et al., 2007), reported by others were not included in this model. For a discussion of the validity of these assumptions and/or how these would affect the model shown in Figure S2, the reader is directed to Scott et al., 2015.

**Figure S3**: Arrow diagrams showing the oxidation of cellobiose, glucose and xylose cellobiose by MnAOx and the hydrolysis of cellobionic acid by β-glucosidase. The oxidation of cellobiose (A), glucose (B) and xylose (C) are described above using a Michaelis-Menten kinetic model with first-order inactivation of the MnAOx. The oxidation of cellobiose to cellobionic acid (G2O) is followed by the hydrolysis of G2O to G and GO by β-glucosidase (D).

The model described in Figure S2 was extended here by including terms that accounted for the oxidation of cellobiose (Figure S3A), glucose (B), and xylose (C) by MnAOx (shortened to AOx in Figure S3). MnAOx activity on each substrate was assumed to follow Michaelis-Menten kinetics and is described using a Michaelis (K_ox_) and catalytic rate constant (*k*_ox_). The superscripts to these constants ^a,b,c^ refer to substrates cellobiose, glucose and xylose, respectively. MnAOx was assumed to be susceptible to first order inactivation to AOx* as described using inactviation rate constant, *k*_i_^b^. In addition, it was assumed that cellobionic acid (G2O) produced from the oxidation of cellobiose by MnAOX was subsequently converted to glucose and gluconic acid (GO) by the action of β-glucosidase (D). The constants that describe the activity of β-glucosidase on cellobionic acid are the Michaelis constant, K_M_^b^, and the cataltyic rate constant, *k*_cat_^b^.

The differential equations that describe the rate of change in cellulose, cellobiose, glucose, cellobionic acid, gluconic acid and xylonic acid are shown below in Equations 1-6, respectively.

Equation 1 $-\frac{\mathrm{dS}}{\mathrm{dt}}=\frac{k_{s} E_{a} S}{S+K_{s}\left( 1+\frac{G}{{K_{G}}^{a}}+\frac{G2}{K_{G2}} \right)}$

where,

S is the substrate cellulose (g/L);

t is time (h);

*k*_s_ is the cellulase catalytic rate constant (gh^-1^g^-1^) (g S x h^-1^ x g E^-1^);

E_a_ is the concentration of active cellulase (g/L);

K_s_ is the cellulase Michaelis-Menten constant (g/L);

G is glucose (g/L)

K_G_^a^ is the competitive glucose inhibition constant for cellulase (g/L)

G2 is cellobiose (g/L)

K_G2_ is the competitive cellobiose inhibition constant for cellulase (g/L)

Equation 2 $\frac{dG2}{\mathrm{dt}}=-\frac{dS}{dt}\times\frac{342 g/mol}{324 g/mol}-\frac{k_{cat}^{a}\mathrm{Bg} G2}{G2+K_{M}^{a}\left( 1+\frac{G}{K_{G}^{b}}+\frac{G2O}{K_{M}^{b}} \right)}-\frac{k_{ox}^{a} \mathrm{AOx}_{a} G2}{G2+K_{ox}^{a}\left( 1+\frac{G}{K_{ox}^{b}}+\frac{X}{K_{ox}^{c}} \right)}$

where,

*k*_cat_^a^ is the β-glucosidase catalytic rate constant (gh^-1^g^-1^) for cellobiose (g G2 x h^-1^ x g Bg^-1^);

Bg is the concentration of β-glucosidase (g/L);

K_M_^a^ is the β-glucosidase Michaelis-Menten constant (g/L) for cellobiose;

K_G_^b^ is the competitive glucose inhibition constant for β-glucosidase (g/L);

K_M_^b^ is the β-glucosidase Michaelis-Menten constant (g/L) for cellobionic acid;

AOx_a_ is the concentration of active aldose oxidase enzyme;

K_ox_^a^ is the MnAOx Michaelis-Menten constant (g/L) for cellobiose.

*k*_ox_^a^ is the MnAOx catalytic rate constant (gh^-1^g^-1^) for cellobiose (g G2 x h^-1^ x g AOx^-1^);

K_ox_^b^ is the MnAOx Michaelis-Menten constant (g/L) for glucose.

K_ox_^c^ is the MnAOx Michaelis-Menten constant (g/L) for xylose.

X is xylose (g/L).

Equation 3 $\frac{\mathrm{dG}}{\mathrm{dt}}=\frac{k_{cat}^{a}\mathrm{Bg} G2}{G2+K_{M}^{a}\left( 1+\frac{G}{K_{G}^{b}}+\frac{G2O}{K_{M}^{b}} \right)}\times\frac{360 g/mol}{342 g/mol}+\frac{k_{cat}^{b}\mathrm{Bg} G2O}{K_{M}^{b}\left( 1+\frac{G2}{K_{M}^{a}} \right)+G2O}\times\frac{180 g/mol}{358 g/mol}-\frac{k_{ox}^{b} \mathrm{AOx}_{a} G}{G+K_{ox}^{b}\left( 1+\frac{G2}{K_{ox}^{a}}+\frac{X}{K_{ox}^{c}} \right)}$

where,

*k*_cat_^b^ is the β-glucosidase catalytic rate constant (gh^-1^g^-1^) for cellobionic acid (g G2O x h^-1^ x g Bg^-1^);

*k*_ox_^b^ is the MnAOx catalytic rate constant (gh^-1^g^-1^) for glucose (g glucose x h^-1^ x g AOx^-1^);

G2O is cellobionic acid (g/L).

Equation 4 $\frac{dG2O}{\mathrm{dt}}=\frac{k_{ox}^{a} \mathrm{AOx}_{a} G2}{G2+K_{ox}^{a}\left( 1+\frac{G}{K_{ox}^{b}}+\frac{X}{K_{ox}^{c}} \right)}\times\frac{358 g/mol}{342 g/mol}-\frac{k_{cat}^{b}\mathrm{Bg} G2O}{K_{M}^{b}\left( 1+\frac{G2}{K_{M}^{a}} \right)+G2O}$

Equation 5 $\frac{\mathrm{dGO}}{\mathrm{dt}}=\frac{k_{cat}^{b}\mathrm{Bg} G2O}{K_{M}^{b}\left( 1+\frac{G2}{K_{M}^{a}} \right)+G2O}\times\frac{196 g/mol}{358 g/mol}$+$\frac{k_{ox}^{b} \mathrm{AOx}_{a} G}{G+K_{ox}^{b}\left( 1+\frac{G2}{K_{ox}^{a}}+\frac{X}{K_{ox}^{c}} \right)}\times\frac{196 g/mol}{180 g/mol}$

where,

GO is gluconic acid (g/L).

Equation 6 $\frac{\mathrm{dXO}}{\mathrm{dt}}=\frac{k_{ox}^{c} \mathrm{AOx}_{a} X}{X+K_{ox}^{c}\left( 1+\frac{G}{K_{ox}^{b}}+\frac{G2}{K_{ox}^{a}} \right)}\times\frac{166 g/mol}{150 g/mol}$

where,

XO is xylonic acid (g/L).

*k*_ox_^c^ is the MnAOx catalytic rate constant (gh^-1^g^-1^) for xylose (g xylose x h^-1^ x g AOx^-1^);

Inactivation of cellulases and aldose oxidase under the assay conditions described above was assumed to be first-order and is described in Equation 7. A performance half-life (t_1/2_) was calculated according to Equation 8. β-glucosidase activity was assumed to be stable.

Equation 7 $\frac{dX_{a}}{\mathrm{dt}}=-k_{i}^{a,b} X_{a}$

where,

X_a_ represents active cellulase (E_a_) or aldose oxidase (AOx_a_)

Equation 8 $t_{1/2}=\frac{0.693}{k_{i}^{a,b}}$

The differential equations were applied to each data set using a 4th order Runge Kutta numerical integration using Microsoft Excel. Each experimental time course was divided into 1000 time steps that increased geometrically from t=0 h. The concentrations of S, G2, G, G2O, GO, X and XO were estimated four times for each time step using Equations 1-6 using the same estimated residual concentration of active cellulase enzyme and MnAOx calculated using Equation 7. The concentration of active cellulase and active MnAOx was recalculated for each time step. The model was used to fit all doses of enzyme tested under a given set of experimental conditions by varying, *k*_s_, *k*_i_^a^, *k*_ox_^a^ and *k*_i_^b^. Optimal values of these parameters were determined simultaneously using the Excel Solver by minimization of least squares. All other parameters in the kinetic model were fixed to the values shown in Additional file Table S3 and S4. 95% confidence intervals and standard deviations of the parameter values were calculated for the cellulase model parameters, *k*_s_ and *k*_i_^a^ each model fit. Student’s T-test was used to determine whether differences in these parameter values between experimental conditions were statistically significant. This information is included in Table 2.

**Table S3** Values for cellulase and β-glucosidase kinetic parameters that were fixed in all model fits.

| Parameter | Value |
| --- | --- |
| K_s_ | 42 g/L |
| K_G_^a^  K_G_^b^ | 13 g/L  1 g/L |
| K_G2_ | 3 g/L |
| *k*_cat_^a^  *k*_cat_^b^ | 100 gh^-1^g^-1^  11 gh^-1^g^-1^ |
| K_M_^a^  K_M_^b^ | 2 g/L  2 g/L |
|  |  |

**Figure S4:** Overlay of new cellulose conversion time course profiles with results published previously (Scott et al., 2016). The cellulose conversion progress curves obtained in this study (closed green diamonds) using a dose of 8.4 mg enzyme protein/g cellulose are shown plotted alongside the data shown previously (open symbols) for CTec3 under these conditions. The doses used previously were 2.8 (blue circles), 5.6 (red diamonds), 8.4 (green squares) and 11.2 (black circles) mg/g. The solid and hatched lines represent the model fit to each data set. The results are plotted as a function of time in panels A, C, E and G. The same data shown in these panels are also shown to the right, plotted and as a function of Enzyme × Time in panels B, D, F, H.

**Figure S5**: Substrate conversion progress curves for CTec3 on pretreated wheat straw when glucose + gluconic acid are both used to calculate the degree of cellulose conversion. CTec3 was incubated with pretreated wheat straw alone (black squares), with catalase (blue diamonds), with MnAOx (green circles) and with MnAOx+catalase (red triangles). Fractional cellulose conversion was in this case calculated using both glucose and gluconic acid. The same experiments in which cellulose conversion is calculated using glucose alone is shown in Figure 4A.

# Effect of Catalase on MnAOx Activity

The gluconic acid profiles shown in Figure 4, panel C under the ambient air condition were analyzed using the model illustrated in Figure S3. The kinetic parameters used to generate the model fits shown in Figure S6 are listed in Table S4. The first row indicates the kinetic parameters determined by Xu *et al.* (2001) for cellobiose and glucose, after conversion to the units used in our model. The calculation of these rate constants for MnAOx are shown below.

*k*_ox_^a^ calculation for cellobiose:

$$k_{ox}^{b}=\frac{12G2}{s}\times\frac{1}{E}\times\frac{mol G2}{6.023\times{10}^{23}G2}\times\frac{342g G2}{mol G2}\times\frac{3600s}{h}\times\frac{6.023\times{10}^{23}E}{mol E}\times\frac{mol E}{55 000g E}={269 gh}^{-1}g^{-1}$$

*k*_ox_^b^ calculation for glucose:

$$k_{ox}^{b}=\frac{4G}{s}\times\frac{1}{E}\times\frac{mol G}{6.023\times{10}^{23}G}\times\frac{180g G}{mol G}\times\frac{3600s}{h}\times\frac{6.023\times{10}^{23}E}{mol E}\times\frac{mol E}{55 000g E}={47 gh}^{-1}g^{-1}$$

The values of the Michaelis-Menten constant of MnAOx for cellobiose (20 g/L) and glucose (8 g/L) as well as the catalytic rate constant of MnAOx for glucose (43 h^-1^) were fixed to these values during the model fits. Kinetic parameters for MnAOx on xylose were not reported previously. However, this enzyme was shown to have a specific activity on xylose that was 76% of the value reported on glucose when assayed using a single substrate concentration. Therefore, the value of the Michaelis-Menten constant of MnAOx was fixed to the same value as glucose (8 g/L) and the catalytic rate constant on xylose was fixed to 33 h^-1^. The model was then fit to the data by varying the inactivation rate constant (*k*_i_^b^) and the catalytic rate constant (*k*_ox_^a^) for MnAOx on cellobiose in the presence and absence of catalase.

The estimates of the model parameters are shown in Table S4. The model suggests that catalase increases both the apparent catalytic rate constant (*k*_ox_^a^) of MnAOx and reduces its inactivation rate constant (*k*_i_^b^). From this one may conclude that catalase increases gluconic acid production in the presence of MnAOx both by reducing H2O2-associated inactivation of the enzyme and by generating oxygen in-situ. However, a relatively small number of data points were used to generate these parameter estimates relative to the complexity of the model. Therefore, a more precise assessment of the relative impact of these two effects of catalase on MnAOx catalysis is not possible given the limitations of the data.

**Figure S6**. Effect of catalase on the formation of gluconic acid by MnAOx under ambient oxygen conditions. Pretreated wheat straw was incubated at 50°C, pH 5 for 96 h under conditions of ambient air (approximately 20% O_2_). The enzyme cocktail consisted of CTec3 + 0.22 mg/ g cellulose MnAOx + catalase (red triangles) and CTec3 + MnAOx (green circles). These data are also shown in Figure 4, panel C. The curves shown in this figure are model fits. The MnAOx model parameters associated with these fits shown in Table S4.

**Table S4.** Kinetic parameters for MnAOx used to model gluconic acid release in CTec3 incubations with pretreated wheat straw in the presence of MnAOx alone (-Cat) or with MnAOx and catalase (+Cat). The gluconic acid data shown in Additional file 2, Figure S6 were fit using the model shown in Additional file 2, Figure S3. When fitting the model to these data, the catalytic (*k*_ox_^a^) and inactivation (*k*_i_^b^) rate constants for MnAOx were varied separately in the absence and presence of catalase while the other parameters were fixed.

|  |  | Cellobiose | | Glucose | | Xylose | |  |  |
| --- | --- | --- | --- | --- | --- | --- | --- | --- | --- |
|  |  | *k*_ox_^a^  (gh^-1^g^-1^) | K_ox_^a^  (g/L) | *k*_ox_^b^  (gh^-1^g^-1^) | K_ox_^b^  (g/L) | *k*_ox_^c^  (gh^-1^g^-1^) | K_ox_^c^ (g/L) | *k*_i_^b^  (h^-1^×10^-2^) | t_1/2_  (h) |
| [18] |  | 269 | 20 | 47 | 8 | - | - | - | - |
| Present Work | -Cat | 484 | 20 | 47 | 8 | 33 | 8 | 2.4 | 29 |
|  | +Cat | 4,033 |  |  |  |  |  | 1.1 | 65 |

# Oxygen use by biomass without mixing, DO rate calculation

Although the biomass under incubation at 40-70°C without mixing drops to <1% DO, the headspace gas was noted to be significantly higher in oxygen composition than the slurry, suggesting that the diffusion rate into unmixed biomass from the headspace is lower than the rate of oxidation within the biomass.

For the 70°C incubated sample, the tube was not opened between cycles, and the headspace oxygen concentration was measured prior to mixing of the substrate and headspace (to oxygenate the substrate for another cycle). The headspace oxygen content, initially approximately 95%, dropped at the end of the incubation cycles from 95% to 74%, 65%, and 55% headspace oxygen in three cycles over 20 hours of incubation; within the limits of this observations, it was calculated that the biomass uses approximately 50 µmole oxygen/g DM/24 hours at 70°C (Table S4). Using the rates as shown in Figure 5, the rate predicted for 50°C would be 12.8 µmole oxygen/g DM/24 hours.

**Table S4** Calculation of oxygen use

| Value | Unit | Description |
| --- | --- | --- |
| 22.4 | liter/mole gas at STP |  |
| 44643 | µmole gas/liter |  |
| 20.95% | %oxygen | 100% air |
| 14.5 | mL | headspace volume |
| 9.35 | µmole/mL | Initial headspace content of oxygen |
| 13.5 | loss in %O2 | average oxygen loss (%O2)/cycle (three cycles) |
| 18 | µmole | average headspace oxygen loss per cycle (three cycles) |
| 95 | %DO | initial %DO biomass |
| 0.7 | %DO | final %DO biomass |
| 16 | mL | biomass volume |
| 261 | µM | predicted solubility of oxygen from air in biomass at 23°C, 5 ppt salinity, 1 atm (1) |
| 4 | µmole | biomass oxygen loss |
| 10 | %TS | dry weight % biomass |
| 1.6 | g DM | dry weigh biomass |
| 42 | µmole/g DM | total oxygen use |
| 20.1 | hours | time at 70°C |
| 50 | µmole/g DM/24 hr |  |

References

1. Garcia, HE and Gordon LI: **Oxygen solubility in seawater**. *Limnol. Oceanogr.,* 1992, **37(6)**:1307-1312.
